# Calculating Reach of Evidence-Based Weight Loss and Memory Improvement Interventions Among Older Adults Attending Arkansas Senior Centers, 2008-2011

**Published:** 2012-02-23

**Authors:** Holly C. Felix, Becky Adams, Jennifer K. Fausett, Rebecca A. Krukowski, T. Elaine Prewitt, Delia Smith West

**Affiliations:** Fay W. Boozman College of Public Health, University of Arkansas for Medical Sciences; Arkansas Department of Health, Little Rock, Arkansas; University of Arkansas for Medical Sciences, Little Rock, Arkansas; University of Arkansas for Medical Sciences, Little Rock, Arkansas; University of Arkansas for Medical Sciences, Little Rock, Arkansas; University of Arkansas for Medical Sciences, Little Rock, Arkansas

## Abstract

**Introduction:**

Older adults could benefit from public health interventions that address the health conditions they face. However, translation of evidence-based interventions into the community has been slow. We implemented 2 evidence-based interventions delivered by lay health educators in Arkansas senior centers from 2008 to 2011: a behavioral weight loss intervention and a memory improvement intervention. The objective of this study was to measure the ability of these programs to reach and serve the growing population of older Americans. We report on differences in program enrollment by age, sex, race, and ethnicity and suggest how our approach to calculating the reach of the 2 interventions can guide future research and program development.

**Methods:**

We defined the reach of the 2 interventions as the proportion of people who needed the intervention and responded to initial recruitment efforts but who did not enroll compared with the proportion of people who needed the intervention and actually enrolled in the program. To calculate these proportions, we used Reach, Efficacy/Effectiveness, Adoption, Implementation, and Maintenance framework formulas. We defined need as the prevalence of obesity (body mass index in kg/m^2^ ≥30) and the level of concern about memory problems among older adults aged 60 years or older. Our target population was 2,198 people aged 60 years or older who attend 15 senior centers in Arkansas.

**Results:**

More than half of our target population responded to recruitment efforts for the behavioral weight loss intervention (61.9%) and for the memory improvement intervention (58.1%), yielding an overall response rate of 59.7%. More than one-third (35.6%) of the target population enrolled in the behavioral weight loss intervention, and 22.8% enrolled in the memory improvement intervention, for an overall reach for the 2 programs of 27.9%.

**Conclusion:**

The reach of 2 evidence-based interventions designed for older adults that targeted specific health conditions and that were delivered in senior centers by community members was high. Our approach to calculating reach in applied settings can guide future research and program development.

## Introduction

Obesity and cognitive impairment are serious issues for older Americans (1-3). Currently, 31.8% of Americans aged 60 years and older are obese (4), and 22.2% have mild cognitive impairment (5). The implementation of evidence-based interventions in community settings to address these conditions may minimize the burdens they impose on health and quality of life; however, translation of such interventions into community practice can be a slow process (6).

Translation research, which studies how to put scientific knowledge into practice, has become a national priority (7). Such research also includes studying the reach of an intervention, that is, the degree to which a target population is represented and served (8). Studying reach has significant public health implications for increasing access to the intervention. The objective of this study was to describe the use of lay health educators (LHEs) to deliver empirically validated weight loss and memory improvement programs in senior centers and focuses on the actual and potential reach of these interventions in the growing population of older Americans. A secondary objective was to describe differences in program enrollment by age, sex, race, and ethnicity.

## Methods

We conducted a clustered randomized controlled trial (NCT-01377506), Translation of Obesity and Cognition Research in a Rural State via Senior Centers, to evaluate the translation of the Lifestyle Weight Loss Intervention (hereafter referred to as the lifestyle intervention) and the Memory Training Intervention (hereafter referred to as the memory intervention) into senior centers. We recruited 16 senior centers in Arkansas and randomly assigned them to 1 of 2 groups, 1 to implement the lifestyle intervention and the other, the memory intervention; 1 senior center withdrew because of staffing issues. Of the remaining 15 centers, 7 were in the lifestyle arm and 8 in the memory arm. The two arms served as controls for each other, requiring all participants to meet the same eligibility criteria. This study design allowed for the evaluation of both of the interventions delivered by LHEs and increased community acceptability by allowing all participating senior centers to offer a program in their community.

We recruited program participants via flyers distributed at senior centers and in the community, news releases, and orientation sessions. At orientation sessions we had attendees complete screening forms to self-assess their potential for program participation. Criteria for selection were age ( ≥60 y), being obese (body mass index [BMI] ≥30 kg/m^2^) but otherwise healthy, ability to walk for exercise, and lack of significant memory problems as determined by a Mini Mental State Exam (11) score greater than 23. These eligibility criteria were imposed for all participants regardless of which of the 2 programs they participated in.

The 2 behavioral interventions were selected for use because they had been proven effective in addressing the health needs of older adults. The lifestyle intervention was adapted from the Diabetes Prevention Program behavioral weight control intervention (9), and the memory intervention was based on the SeniorWISE Memory Improvement Program (10). We trained LHEs to deliver the programs, which were then offered in the senior centers in 12 weekly group sessions, followed by 9 monthly group sessions.

Initial analyses of primary study outcomes (weight change and memory improvement) indicated that both programs that LHEs delivered were effective in producing weight loss and memory improvement at 4 months, compared with their controls. More than one-third (38%) of older adults in the lifestyle intervention experienced a clinically meaningful weight loss of 5% or more of their baseline weight at 4 months compared with only 5% of those in the control arm (P <.001) (13). Similarly, 33% of older adults in the memory intervention experienced a clinically meaningful improvement in delayed memory compared with 17% of those in the control arm (14).

### Data sources

For our evaluation of reach, we used data on the number and characteristics of people screened and enrolled or excluded from the study that were collected by the LHEs and study staff during the trial's recruitment phase. Additionally, we obtained data on characteristics of older adults in Arkansas and the United States from the 2008 National Aging Program Information Systems (NAPIS) State Program Reports (15). We derived characteristics of older adults who attend US senior centers from the 2004 Administration on Aging (AOA) Survey of Older Americans Act Participants (16). The most recent (2004) AOA survey targeted only participants in the daily congregate meal program, not all older adults who attend senior centers. Because the meal program draws most older adults to senior centers and because the last known survey of all participants was in 1984, the 2004 AOA survey was considered to be a reasonable resource to characterize all senior center participants. We used National Health and Nutrition Examination Survey data to estimate the prevalence of obesity among older Americans (17) and National Council on Aging/MetLife Foundation survey results to estimate the potential audience for a memory intervention based on the number of adults 60 years and older whom the survey indicated were worried about memory loss (18).

### Population

In 2007, the year prior to senior center recruitment for our study, 561,850 Arkansas residents were aged 60 years or older. They were predominantly female (55.9%) and white (87.1%) (15). In that same year, the 184 senior centers in Arkansas had 26,963 adults participating in congregate meal programs, an average of 146.5 adults per senior center (16). Therefore, we estimated that 2,198 adults attended the 15 senior centers recruited to our study from which we drew our target population. Given that obesity prevalence is 31.8% among older adults (4), we estimated that 326 older adults attending the 7 senior centers randomly assigned to the lifestyle intervention were obese and represented the target population for the lifestyle program. Approximately 42% of older adults express concern about memory loss (17). Therefore, an estimated 492 older adults attending the 8 senior centers assigned to the memory arm of our study would potentially be interested in a memory program and represent the target population for the memory program.

### Statistical analysis

The analysis of program reach was guided by the RE-AIM (Reach, Efficacy/Effectiveness, Adoption, Implementation, and Maintenance) framework, which was designed to assess the effect of health interventions at the individual and organizational levels and across research phases (ie, recruitment, implementation, and post implementation) (18-21), and we followed steps outlined by the National Cancer Institute's Implementation Science website (8). Actual reach was calculated as the proportion of individuals in need of the intervention who were engaged during recruitment compared with the proportion of individuals in need of the intervention who actually enrolled. Potential reach was estimated using that method, but assuming implementation across all senior centers in Arkansas and the nation.

Descriptive statistics characterized individuals screened for study participation. We compared demographics for seniors who enrolled and did not enroll using generalized linear models or generalized estimating equations, which accounted for the clustering of seniors in senior centers. For those who enrolled, we used SAS version 9 (SAS Institute, Inc, Cary, North Carolina) to compute proportions and corresponding 95% confidence intervals using Taylor series variance estimation to account for clustering. Comparisons with the larger target population were deemed significant (*P* < .05) when the national survey estimate fell outside the corresponding limits.

The institutional review board of the University of Arkansas for Medical Sciences approved all study procedures.

## Results

### Recruitment

A total of 488 seniors, 202 in the lifestyle intervention and 286 in the memory intervention, responded to recruitment efforts by attending orientation. On the basis of our review of the self-assessment screening forms, we determined that 84% (n = 410 [lifestyle, n = 181; memory, n = 229]) were likely eligible, and we referred them into the full study screening process. The primary reasons for ineligibility were based on self-screener responses related to body weight or ability to walk for exercise, criteria we imposed to ensure the safety and efficacy of the lifestyle intervention.

### Enrollment

Of the 410 seniors eligible for screening for the full study, 349 (85.1%) were screened, 161 for the lifestyle intervention and 188 for the memory intervention. Sixty-one were not screened because they could not be contacted on follow-up or because they refused to complete the screening interview when contacted. There were no significant differences in the reasons for ineligibility by intervention arm during this screening process. Of people who were screened, 228 were fully eligible to participate and were enrolled (116 in the lifestyle study arm and 112 in the memory arm). Three people who were eligible declined to enroll in the study. The primary reason for exclusion during the full screening process was a BMI below 30, followed by failure to complete the screening process, and this pattern was evident in both study arms (Table 1). There was a significant difference between eligible and noneligible people by race (*P* = .03); 56.1% of nonwhite seniors were ineligible compared with 31.8% of whites. The primary reason that nonwhites were excluded was failure to complete the screening process (47.8%) followed by recent weight loss or history of weight loss surgery (17.4%). The primary reason that whites were excluded was a BMI below 30 (51%), followed by failure to complete the screening process (22.4%). There were no significant differences in age, sex, or race between those enrolled (n = 228) and those excluded (n = 121) (Table 2).

For the lifestyle intervention, 202 people (69% of the target population of 326) responded to recruitment efforts. Nearly all who were initially eligible (99.1%) continued with the screening process, were determined to be fully eligible for participation, and enrolled in the program (n = 116). Therefore, we estimate that the lifestyle intervention had a reach of 35.6% (116 of 326) in the target population. Lifestyle participants were younger (mostly aged 60-74 y), and a larger percentage were women (91.4%) than in the target population overall.

## Discussion

The approximately 11,000 senior centers in the United States serve 1,656,330 older adults (15). Given current obesity prevalence estimates of 31.8% among adults aged 60 or older (4), approximately 526,713 older Americans attending senior centers are likely to be obese. Were the 35.6% reach into the target population that was achieved in the current study extended to the larger population of obese older adults attending senior centers, an estimated 187,510 seniors in need of such a program could be reached.

At senior centers participating in the study's memory arm, 286 seniors, or 58.1% of the target population (n = 492), responded to recruitment activities, and 112 enrolled in the study. Therefore, we estimate that the memory intervention had a reach of 22.8% in the target population. Of the people excluded during the full screening process, 91 were excluded on the basis of lifestyle intervention criteria (eg, minimum BMI, ability to walk for exercise). These criteria may not be required for a memory intervention implemented in the community and outside the unique constraints of our study's design. Therefore, it could be estimated that without being subject to the lifestyle study's eligibility criteria, the memory intervention's reach could have been as high as 41.3%. As with lifestyle participants, participants in the memory intervention differed significantly from our target population with respect to age but were similar in sex and race. Most memory program participants were also younger, between 60 and 74 years.

Using current estimates of the number of older adults who are concerned about memory loss (17), an estimated 695,659 (42%) of older adults attending US senior centers would likely be interested in the memory intervention. On the basis of our memory intervention's reach of 112 seniors (22.8% of our target audience of 492), we estimate that 158,610 older adults would benefit from such a program if the memory intervention were translated into all US senior centers. Furthermore, the potential reach of the memory intervention is likely greater than the target population penetration achieved in this study suggests because memory program participation was constrained by the exclusion of nonobese people who were interested in the program. Under normal program delivery conditions, older adults with memory concerns would not be excluded from a memory intervention for lifestyle intervention criteria such as obesity. If current reach estimates are revised to include those seniors who were excluded from the memory program on the basis of the lifestyle intervention's exclusion criteria (n = 91), we would have reached a total of 203 people or 41.3% of our target population of 492. This extrapolates to a larger pool of 287,307 older adults likely to participate and benefit from the memory intervention if it were implemented nationally.

Approximately two-thirds of our target population responded to recruitment efforts for the 2 evidence-based health promotion programs, and more than one-fourth of the target population enrolled in the programs. These estimates of reach are likely conservative because they do not include people who self-selected out of the screening process because they thought they were ineligible or because they were not interested in being research participants. Nevertheless, our reach estimates indicate a high receptivity among older adults for community-delivered programs targeting weight loss and memory improvement. Efforts have been made to evaluate interventions demonstrated efficacious in clinical trials that are implemented in real-world settings to benefit community-dwelling older adults (22). However, comparing the reach into the target population achieved in our study with the reach reported by other studies translating similar interventions into community settings (23-24) is difficult, because other studies primarily report the proportion of individuals screened who enrolled rather than the proportion of the target population reached following application of the RE-AIM framework (18-21). The proportion of individuals screened for eligibility and then enrolled in those studies averages approximately 16% (23-24), which is lower than what we found. For example, the DEPLOY study (23) was a cluster-randomized trial undertaken to translate the Diabetes Prevention Program lifestyle intervention into YMCAs. That study screened 535 adults but enrolled only 92 (17.2%), whereas our study screened 349 older adults and enrolled 228 (65%). However, differences in eligibility criteria (DEPLOY required impaired glucose tolerance, whereas our study did not) may have contributed to the discrepancy in reach.

One advantage to calculating reach using the RE-AIM framework rather than limiting reach estimates to recruitment yields is that projected reach using the broader target population more precisely informs policy-makers about the potential to serve the needs of their target community. Our estimates of a 27.9% reach into a population of older adults who are obese and experience memory concerns coupled with evidence that LHE-delivered programs can be effective in translating public health interventions to older adults (approximately one-third of participants in the study experienced clinically meaningful outcomes [13-14]) are timely. National health care reform efforts include the expansion of home- and community-based services for older adults and support use of community health workers (eg, LHEs) (23). Federal research is focusing on the translation of research to support health and independence of community-dwelling older adults (26).

Our study has limitations. Calculating the size and characteristics of the target population was challenging because the availability of data describing older adults attending US senior centers is limited. Our analysis relied on 2 surveys of congregate meal program participants to define the target population and assess the degree to which study participants were representative of the target population. Because senior centers offer a variety of programs and services beyond their congregate meal program, data on the subset of older adults in the congregate meal program may not adequately characterize senior center attendees overall. Participants in the congregate meal program may be older and more infirm than those seniors participating in other senior center programs. Participants in our study were younger and included more women than the target population defined by surveys of congregate meal participants. More recent and comprehensive data on individuals who frequent senior centers would allow a better estimate of the target population and thus a more precise estimate of the program reach. Additionally, our analysis was restricted to older adults attending senior centers and not the larger older-adult population. Senior centers serve a small proportion of all older adults in the community. For example, in Arkansas, approximately 5% of older adults attend senior centers. However, offering evidence-based programs for weight management and memory improvement may stimulate attendance at senior centers by older adults who would not otherwise have attended, given the interest in memory and weight loss among older adults. Indeed, 23% of study participants were infrequent (fewer than 1 visit per month) users of the senior centers.

To meet the preferences and activity levels of the large emerging population of older adults arising from the aging of the Baby Boom generation, many US senior centers are being transformed into multipurpose centers that offer more services, including health and wellness programs (27). The provision of evidence-based health and wellness programs by senior centers is also being encouraged by the federal government (28). The lifestyle and memory interventions implemented in this study are 2 such programs. Our results indicate these programs have the potential to reach a substantial number of older Americans. Furthermore, participants in this study were younger than those in the target population of senior center attendees, suggesting that the memory and lifestyle programs may well be appealing to the Baby Boom generation.

Understanding a program's reach is critical when making decisions on funding and implementing health programs; knowing how many people in the target population will participate in and benefit from a program is a key consideration for allocating scare resources. Our analysis demonstrates that the reach of 2 evidence-based programs in memory improvement and weight control tailored for older adults and delivered by LHEs in senior centers is substantial.

## Acknowledgments

This study was supported in part by grant no. R18 DP001145 from the Centers for Disease Control and Prevention. Additional support for Dr Felix came from a career development award (KL2RR029883) from the University of Arkansas for Medical Sciences Translational Research Institute (National Center for Research Resources award no. 1UL1RR029884). The authors acknowledge the contributions of participating senior centers and the Arkansas Department of Health, which made this work possible, and the assistance of Shelley Lensing, MS, Sharhonda J. Love, MPH, and Debbie Hodges, for their assistance with data processing and analysis.

## Figures and Tables

**Table 1:** Enrollment and Exclusion by Study Arm and by Race of Older Adults Screened for Participation in Weight Loss and Memory Improvement Interventions in Arkansas Senior Centers, 2008-2011

Characteristic	Total (N = 349)	Study Arm		Race	

		Lifestyle (n = 161)	Memory (n = 188)	**White (n = 308)**	Nonwhite (n = 41)

		n (%)			
Enrolled	228 (65.3)	116 (72.0)	112 (59.6)	210 (68.2)	18 (43.9)
Excluded	121 (34.7)	45 (28.0)	76 (40.4)	98 (31.8)	23 (56.1)^a^
**Reasons for exclusion**					
Eligible, did not enroll	3 (2.5)	1 (2.2)	2 (2.6)	2 (2.0)	1 (4.3)
BMI <30 kg/m^2^	53 (16.1)	21 (46.7)	32 (42.1)	50 (51.0)	3 (13.0)
MMSE ≤23	5 (1.5)	1 (2.2)	4 (5.3)	3 (3.1)	2 (8.7)
Recent 10% WL or WL surgery	10 (3.0)	5 (11.1)	5 (6.6)	6 (6.1)	4 (17.4)
Study cohort full	4 (1.2)	4 (8.9)	0	4 (4.1)	0
Failed to complete screening	33 (10.0)	7 (15.6)	26 (34.2)	22 (22.4)	11 (47.8)
Other^b^	13 (4.0)	6 (13.3)	7 (9.2)	11 (11.2)	2 (8.7)

Abbreviations: BMI, body mass index; MMSE, Mini Mental State Examination; WL, weight loss

a *P* = .03. *P* values generated using generalized estimating equations.

b Time conflict, family disapproval or conflict, no longer interested.

**Table 2:** Demographic Characteristics of Older Adults Screened for Participation in Weight Loss and Memory Improvement Interventions in Arkansas Senior Centers, by Enrollment Status, 2008-2011^a^

**Characteristic**	All Screened (n = 349)	Enrolled (n = 228)	Not Enrolled (n = 121)	*P* Value^b^
**Age at screening, y**				
Mean (SD)	71.6 (6.9)	71.2 (6.6)	72.4 (7.4)	.20
Range	60-89.4	60-87.2	60.4-89.4	
**Sex**				
Male	58 (16.6)	36 (15.8)	22 (18.2)	.49
Female	291 (83.4)	192 (84.2)	99.(81.8)	.49
**Race**				
White	308 (88.3)	210 (92.1)	98 (81.0)	.03
Black or African American	39 (11.2)	18 (7.9)	21 (17.4)	.03
American Indian/Alaska Native	2 (0.6)	0 (0)	2 (1.7)	.03
**Ethnicity**				
Hispanic	3 (0.9)	1 (0.4)	2 (1.7)	.27
Non-Hispanic	346 (99.1)	227 (99.6)	119 (98.3)	.27

Abbreviation: NC, not calculated.

a All values expressed as no. (%), unless otherwise indicated.

b *P* values generated using generalized estimating equations.
